# Peripheral vascular remodeling during ischemia

**DOI:** 10.3389/fphar.2022.1078047

**Published:** 2022-12-01

**Authors:** Shigang Lin, Ruoran Lin, Hongkun Zhang, Qingbo Xu, Yangyan He

**Affiliations:** ^1^ Department of Vascular Surgery, The First Affiliated Hospital, Zhejiang University School of Medicine, Hangzhou, China; ^2^ Department of Cardiology, The First Affiliated Hospital, Zhejiang University School of Medicine, Hangzhou, China

**Keywords:** ischemia, endothelial cells, smooth muscle cells, fibroblasts, vascular disease

## Abstract

About 230 million people worldwide suffer from peripheral arterial disease (PAD), and the prevalence is increasing year by year. Multiple risk factors, including smoking, dyslipidemia, diabetes, and hypertension, can contribute to the development of PAD. PAD is typically characterized by intermittent claudication and resting pain, and there is a risk of severe limb ischemia, leading to major adverse limb events, such as amputation. Currently, a major progress in the research field of the pathogenesis of vascular remodeling, including atherosclerosis and neointima hyperplasia has been made. For example, the molecular mechanisms of endothelial dysfunction and smooth muscle phenotype switching have been described. Interestingly, a series of focused studies on fibroblasts of the vessel wall has demonstrated their impact on smooth muscle proliferation and even endothelial function via cell-cell communications. In this review, we aim to focus on the functional changes of peripheral arterial cells and the mechanisms of the pathogenesis of PAD. At the same time, we summarize the progress of the current clinical treatment and potential therapeutic methods for PAD and shine a light on future perspectives.

## Introduction

Peripheral arterial disease (PAD) is a kind of vascular disease in which the blood flow is restricted due to stenosis or obstruction of arteries, resulting in tissue ischemia. There are various disease included in PAD, such visceral artery aneurysm, arteritis, carotid artery stenosis and atherosclerosis obliterans (ASO). In this review, PAD mainly refers to ASO. With increasing incidence and mortality, PAD has affected more than 230 million people worldwide (Song et al., 2020). Traditional cardiovascular risk factors, including advanced age, hypertension, diabetes, and smoking, are strongly associated with the risk of PAD (Selvin and Erlinger, 2004; Allison et al., 2006; Fowkes et al., 2013; Whelton et al., 2018). Patients who suffer from PAD often have coronary or cerebrovascular disease as well. The typical symptom of PAD is intermittent claudication, mainly being manifested in the lameness of the lower limbs after walking for a certain distance. The above symptoms can be relieved by a short rest (Gerhard-Herman et al., 2017; Golledge, 2022). As the disease worsens, the patient develops resting pain (Jones and Farber, 2020). This is due to the aggravation of the lesion, resulting in occlusion of arteries, leading to ischemia and hypoxia of the skeletal muscle. After tissue ischemia, peripheral arteries display a positive enhancement of vascular remodeling, in which atherosclerosis is the main cause of peripheral vascular obstruction (Newman et al., 1993; Stock, 2022). On the other hand, ischemia can also trigger a series of cellular events that lead to peripheral vascular remodeling.

At the present, the exploration of the pathogenesis of peripheral artery disease is limited. We do not yet fully understand the complex cellular functional changes and the mechanisms underlying cell-to-cell interactions that drive PAD. Under physiological conditions, vascular cells, such as endothelial and smooth muscle cells (SMCs), and fibroblasts, are essential for maintaining stable vascular structure and function. When ischemia occurs, dysfunction of vascular cells leads to peripheral arterial remodeling. Currently, drug usage and revascularization approaches are the main treatments for PAD (Wiseman et al., 2017; Farb et al., 2021; Sogaard et al., 2021), although the effectiveness is limited. In this article, we aim to discuss the mechanism of peripheral vascular cell dysfunction under ischemic conditions, summarize the clinical treatment and potential therapeutic methods and highlight the potential future research focusing on PAD.

### The role of endothelial cells in peripheral arterial disease

Vascular endothelium regulates the cellular and molecular transport between blood and tissues and plays an important role in maintaining the stability of vessel wall (Bazzoni and Dejana, 2004; Trimm and Red-Horse, 2022). Tissue ischemia leads to lower oxygen supply, free radical production, and other cellular response in most tissues. In response to tissue ischemia of the limb, the inflammatory response of endothelial cells (ECs) is closely related to the progression of vascular lesion development (Gimbrone and Garcia-Cardena, 2016; Duan et al., 2021). As a critical enhancer, tissue ischemia not only induced the expression of Vcam-1 and Icam-1 on ECs, but also upregulated the levels of inflammatory markers (IL-1β, IL-6 and TNF-α). Meanwhile, tissue ischemia also results in production of platelet-derived exosomes in serum that play an important role in a variety of pathological mechanisms (Li et al., 2017). Exosomes are mainly involved in immune response and cell-to-cell communication in the pathogenesis of atherosclerosis (Bu et al., 2021). As an intercellular communication carrier, exosomes can wrap miRNA for transfer. It has been found that miRNA regulate the expression of related target genes by matching the target sites of the 3′untranslated region (3′utr) of target genes (Ambros, 2004; Bartel, 2004). Evidence showed that ECs could endocytose exosomes to upregulate miR-25-3p, and suppress levels of α-smooth muscle actin (α-SMA), collagen I a1, Collagen III a1, IL-1β, IL-6, and TNF-α. miR-25-3p inhibits endothelial inflammation by inhibiting the expression of Adam10, and reducing the phosphorylation level of NF-kB signaling pathway (Yao et al., 2019). Exosomes containing miR-155 have been reported to transmigrate from smooth muscle cells (SMCs) to ECs, causing endothelial dysfunction and ultimately aggravating lesion formation of atherosclerosis (Zheng et al., 2017). Furthermore, miR-155 ameliorates ox-LDL-induced inflammation in human umbilical vein endothelial cells (HUVECs) by inhibiting SOCS1-dependent NF-kB signaling (Zhang et al., 2017), in which miR-19 attenuates endothelial dysfunction induced by ischemia by negatively regulating KLF10 and inhibiting TGF-β1/Smad signaling pathway (Xu et al., 2018). In addition, Jiang et al. (2019) found that miR-449a was significantly up-regulated in atherosclerosis lesions and also was able to promote proliferation and migration of ECs. Besides, it could also induce expression of α-SMA and Smad3, and inhibits E-cadherin expression in ECs. Eventually, it was found that miR-449a enhances the expression of VACM-1 and ICAM-1 induced by TNF-α in ECs (Jiang et al., 2019). These results indicate that miRNA is an important regulator of endothelial inflammation in response to ischemia, where exosomes are one of the important carriers for miRNA transport.

**FIGURE 1:**
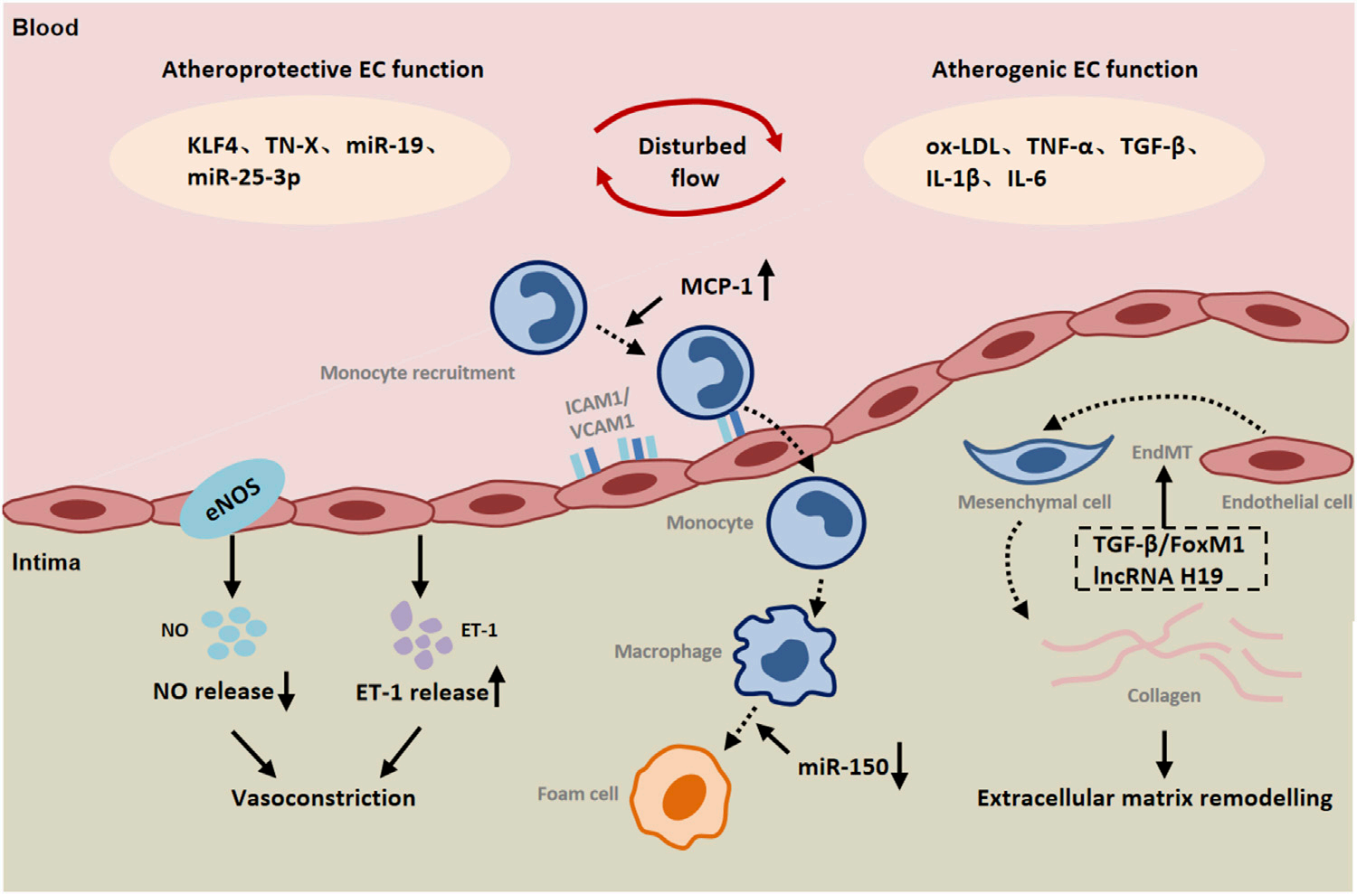
The role of endothelial cells (ECs) in peripheral arterial disease (PAD). Both atheroprotective and atherogenic mechanisms are operative in endothelial cells (ECs) exposed to disturbed flow. Endothelial cells are involved in communication with immune cells and smooth muscle cells by releasing a variety of inflammatory cytokines, chemokines and ET-1. Endothelial-to-mesenchymal transition (EndMT) contributes to vascular remodeling. Abbreviations: KLF4, Kruppel like factor 4; TN-X, Tenascin-X; oxLDL, oxidized low-density lipoprotein; IL-1β, interleukin 1 β; IL-6, interleukin 6; TGF-β, transforming growth factor-β; ICAM-1, intercellular cell adhesion molecule-1; VCAM-1, vascular cell adhesion molecule-1; MCP-1, monocyte chemotactic protein-1; FoxM1, Forkhead Box M1; ET-1, endothelin-1.

Ischemia-induced EC dysfunction can also lead to the overexpression of endothelin-1 (ET-1). As a potent vasoconstrictor, endothelin-1 (ET-1) is mainly secreted by ECs and interacts with two different G-protein-coupled receptors, ETA and ETB (Yanagisawa et al., 1988; Miyauchi and Masaki, 1999). SMCs express ETA and ETB receptors to mediate vasoconstriction, whereas ETB receptors on ECs mediate vasodilation through the release of nitric oxide (NO) and prostacyclin (Kirchengast and Munter, 1998; Luscher and Barton, 2000). ET-1 has been reported to play an important role in neointimal formation and atherosclerosis (Bacon et al., 1996; McKenna et al., 1998). ET-1 expression is upregulated in diseased vessels when stimulated by shear stress. It was found that loss of ET-1 was associated with decreased expression of VCAM-1 and MCP-1 in endothelial cells and decreased recruitment of inflammatory cells to the vessel wall (Anggrahini et al., 2009). *In vitro* studies also showed that ET-1 enhanced the expression of VCAM-1 in TNF-α-stimulated ECs (Ishizuka et al., 1999). ET-1 has been reported to induce proliferation of SMCs through ERK, p38-activated protein kinase and c-MyC-dependent signaling (Chen et al., 2006). ETA receptor signaling mediates SMC proliferation (Hafizi et al., 1999). Numerous studies revealed that ETA and ETB expression were decreased and SMC proliferation was attenuated in ET-1 null diseased vessels (Anggrahini et al., 2009). These results suggest that EC-derived endothelin-1 is involved in mediating SMCs proliferation and endothelial inflammation in a paracrine manner, thereby promoting vascular remodeling (Figure 1).

Moreover, inflammation can induce endothelial-to-mesenchymal transition (EndMT), which leads to pathological vascular remodeling (Cho et al., 2018; Sanchez-Duffhues et al., 2019). EndMT is a phenomenon that ECs lose their endothelial markers and acquire mesenchymal characteristics. During EndMT, endothelial markers such as VE-cadherin and CD31 are lost, while the expression of mesenchymal markers such as fibroblast specific protein-1 (FSP-1), α-SMA, N-cadherin, and fibronectin is increased (Li et al., 2018; Kovacic et al., 2019). TGF-β is a key mediator of EndMT. Studies have shown that TGF-β promotes the phosphorylation of Smad2/3, and then regulates the expression of Snail, Twist, Slug and other transcription factors and promotes EndMT (Medici et al., 2011). Recently, increasing evidence highlights the potential role of Foxm1 in vascular fibrosis/remodeling (Balli et al., 2013). According to the report, TGF-β could induce the expression of Foxm1 in ECs. Foxm1 promoted the expression of TGF-β-induced vimentin, α-SMA and FSP1 in ECs through Smad2/3 signaling pathway, and inhibited the expression of VE-cadherin and CD31 (Song et al., 2019). FoxM1 has been reported to interact with Smad3 to maintain activation of the Smad2/Smad3/Smad4 complex in the nucleus. The complex translocated to the nucleus and activated the expression of ECM-related genes as well as Snail, and Twist genes (Xue et al., 2014; Sabbineni et al., 2018). It has been found that Snail inhibits VE-cadherin expression by directly binding to the E-box sequence within the VE-cadherin promoter (Cano et al., 2000). Activation of AMP-activated protein kinase (AMPK) upregulates the level of peroxidase proliferator-activated receptor-γcoactivator-1α (PGC1α), resulting in a decrease in phosphorylated Smad2 and the nuclear translocation of Snail, thereby inhibiting EndMT (Mao et al., 2022). The intracellular signaling pathway mediated by protein kinase Akt is closely related to the regulation of endothelial cell survival, proliferation, migration, glucose metabolism and gene expression (Shiojima and Walsh, 2002). Here, it was found that silencing Akt1 in endothelial cells resulted in increased expression of mesenchymal genes (e.g FN1 and KRT7) and decreased expression of eNOS, accompanied by activation of p38 MAP kinase. AKT1 can negatively regulate the expression of TGFβ2 and Smad2/3 phosphorylation, as well as the expression of Snail1 and FoxC2 (Sabbineni et al., 2019). In addition to the expression of mesenchymal markers, EndMT is also characterized by changes in EC morphology (Krenning et al., 2016). It was observed that normal ECs had a polygonal cobblestone shape, whereas AKT1-null ECs changed into a more spindle-like fibroblast shape. Inhibition of β-catenin reverses EndMT induced by loss of Akt1 (Sabbineni et al., 2019). Taken together, ischemia can directly and indirectly lead to endothelial-to-mesenchymal transition that plays a part in vascular remodeling in peripheral arteries.

**FIGURE 2:**
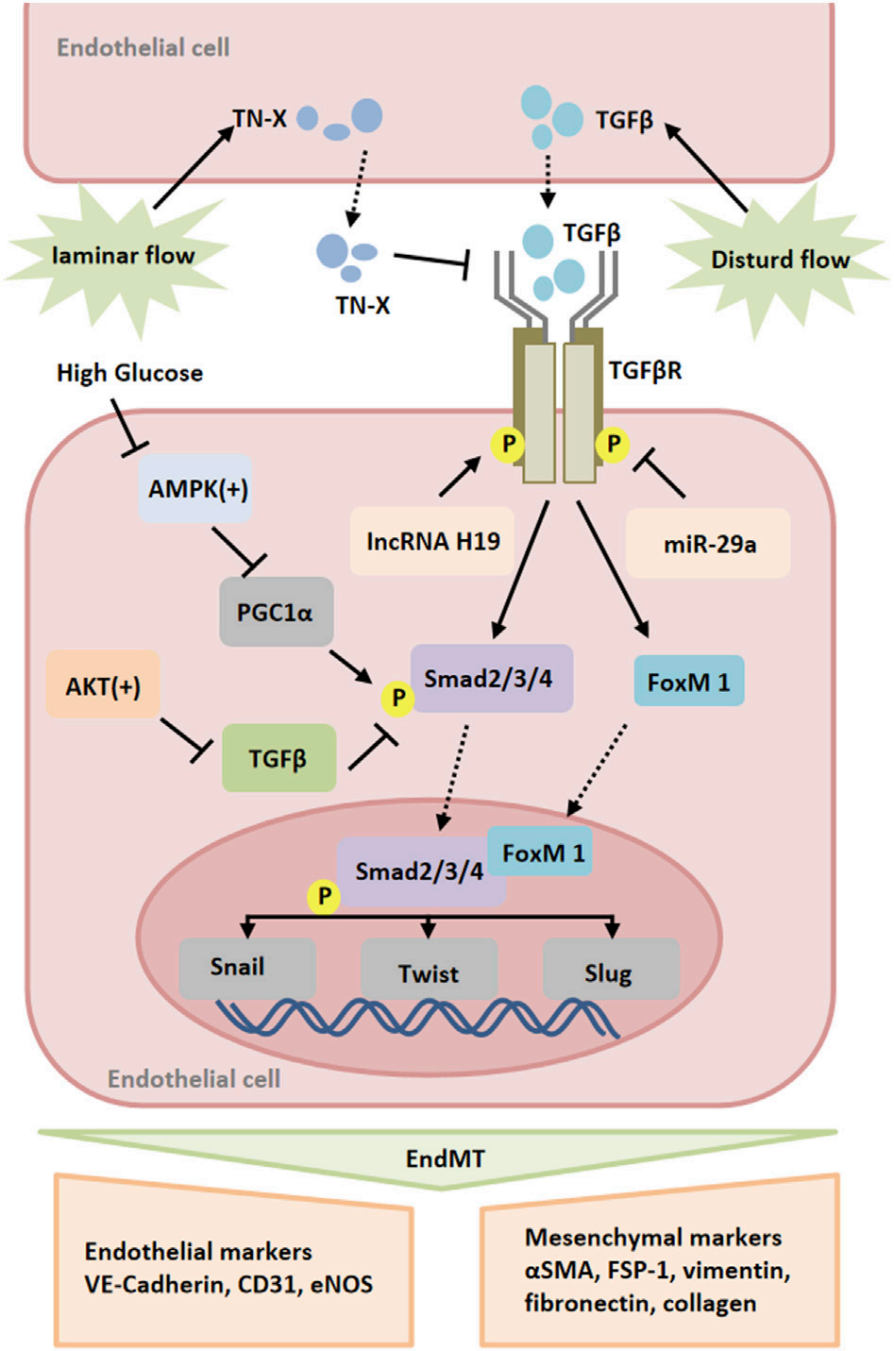
Signaling pathways of endothelial-to-mesenchymal transition (EndMT). Multiple stimuli, such as TGF and high glucose, induce the expression of transcription factors, such as Snail, Slug and Twist, which promote EndMT. These processes are regulated by various mediators, such as miR-25-3p, lncRNA H19, Smad2/3/4, FoxM1, AMPK and AKT. Abbreviations: AMPK, 5′-adenosine monophosphate (AMP)-activated protein kinase; FoxM1, Forkhead Box M1.

It has been found that different forms of blood flow activate different signal transduction pathways in ECs, leading to vascular remodeling (Zhou et al., 2014; Nakajima and Mochizuki, 2017). In terms of peripheral arteries during ischemia, often there is atherosclerotic lesions resulting altered blood flow. Disturbed flow induces endothelium mesenchymal transition (EndMT). Unidirectional laminar flow inhibited TGF-β signaling as well as TGF-β-induced EndMT compared with disturbed flow (Moonen et al., 2015; Lee et al., 2017). The study found that laminar flow increased endothelial TN-X expression in a KLF4-dependent manner. TN-X expression in endothelial cells was negatively correlated with the expression of mesenchymal marker genes and inflammatory genes, such as FN1, CCL2, VACM-1, and the phosphorylation of Smad. TN-X directly interacts with TGF-β through its fibrin-like domain, thereby interfering with its binding to TGF-β receptors (Liang et al., 2022). Elevated TGF-β levels were also found in the plasma of TN-X deficient patients (Morissette et al., 2014). In addition, lncRNA H19 was significantly upregulated in TGF-β-induced EndMT. The expression of endothelial marker CD31 was up-regulated, and the inhibition of mesothelial cell marker FSP-1 was related to the inhibition of lncRNA H19. Moreover, the increased expression of lncRNA H19 could alleviate the inhibitory effect of miR-29a on EndMT-related gene expression (Shi et al., 2020). These results indicate that lncRNA H19 can compete with mir-29a and participate in the regulation of EndMT development through TGF-β/SMAD3 pathway (Figure 2).

Tissue ischemia results in accumulation of free radicals locally, especially in peripheral arteries. In fact, oxidative stress is one of the important causes of vascular endothelial dysfunction (Cai et al., 2003). Elevated vascular reactive oxygen species (ROS) production is considered to be an early trigger mechanism for endothelial dysfunction, including oxidative stress, activation of proinflammatory signaling cascades, and reduction of endothelial nitric oxide (NO) bioavailability (Hsieh et al., 2014; Blaser et al., 2016; Wu et al., 2018). The NOX protein family contains several isoforms, including NOX1, NOX2, and NOX4, whose sole function is to produce ROS (Bedard and Krause, 2007). Under physiological conditions, NOX1 and NOX2 are expressed at low levels in the peripheral vascular, but up-regulated in pathological situations such as hypertension, diabetes and hyperlipidemia (Mollnau et al., 2002; Wendt et al., 2005). Increased NOX1 expression in ECs promotes the expression of MCP-1, VCAM-1, CTGF and collagen I/III/IV, which contributes to lesion formation (Gray et al., 2013). Moreover, there was a study demonstrates that NOX2 expression was upregulated in ECs of diseased vessels. Further studies revealed that decreased arterial superoxide production could significantly reduce lesion formation and increased NO bioavailability when NOX2 was deficient (Judkins et al., 2010). Endothelium-derived NO plays a protective role in vasculature by inhibiting the contraction and proliferation of vascular SMCs. NOX derived ROS can not only reduce the bioavailability of NO, but also directly react with NO to produce the oxidizing substance peroxynitrite (Judkins et al., 2010). Peroxynitrite has been reported to oxidize tetrahydrobiopterin (BH4), a cofactor of eNOS, leading to uncoupling of eNOS (Laursen et al., 2001). Compared with NOX1 and NOX2, NOX4 mainly plays a protective role in the cardiovascular system. NOX4 is predominantly expressed in ECs. Multiple recent reports indicate that Nox4 produces primarily H_2_O_2_ rather than superoxide (Schader et al., 2020). Low concentration of H_2_O_2_ contributes to vascular protection. H_2_O_2_ can not only maintain vasodilatation by activating protein kinase G, but also activate and induce eNOS expression (Indra et al., 1999; Garcia-Prieto et al., 2019). It has been found that H_2_O_2_ promoted vasodilation by enhancing Ca2+ release from endoplasmic reticulum stores, which in turn promoted the opening of Ca2+ -activated K+ channels (Hecquet et al., 2008). Katrin et al. proved that endogenous Nox4 accelerated the expression of eNOS, the formation of NO, and the expression of the antioxidant HO-1. HO-1 expression is controlled by the transcription factor Nrf-2. NOX4 reduces Nrf-2 degradation by producing H_2_O_2_, thereby promoting HO-1 expression. Loss of NOX4 not only exacerbates Ang II-induced vascular stenosis but also increases the rate of endothelial cell apoptosis, which is an important stimulator of vascular inflammation (Schroder et al., 2012). Moreover, NOX4 in endothelial cells can inhibit the expression of MCP-1, IL-1β, TGF-β and other proinflammatory factors, and limit vascular inflammation and macrophage infiltration. It has been reported that TGF-β/SMAD3 signaling pathway is involved in the regulation of CTGF expression and vascular fibrosis (Mao et al., 2020). The expression of EC-NOX4 was negatively correlated with the phosphorylation level of SMAD3 and the expression of CTGF (Gray et al., 2016). Thus, the balance between NOX-mediated free radical generation and eNOS pathway-generated NO production can be disturbed during ischemia, resulting endothelial dysfunction and eventually peripheral vascular remodeling.

### The role of smooth muscle cells in peripheral arterial disease

In normal arteries, SMCs remain quiescent and highly differentiated. However, they have dedifferentiation potential and plasticity compared with skeletal muscle and cardiomyocytes. Under pathological conditions, SMCs can switch from a contractile to a synthetic phenotype, which is also known as SMC phenotypic switching. During SMC phenotypic transition, the cell loses specific markers, such as smooth muscle actin and SMMHC (Hao et al., 2003; Gomez and Owens, 2012). This type of SMCs serves as main player for intima hyperplasia that is a key event for peripheral vascular remodeling (Owens et al., 2004). In response to ischemia and ischemia-mediated cytokines released from ECs, SMC undergo metabolic reprogramming upon activation to rapidly provide high energy and meet the biosynthetic requirements for cell proliferation (Kim et al., 2017). Increased glycolysis is essential for bioenergetic transfer during SMC proliferation and migration (Park et al., 2021). Pyruvate kinase (PKM2) is an important regulatory element in glycolysis and is mainly expressed in highly proliferating cells, such as stem cells and tumor cells (Xie et al., 2016; Liu et al., 2017). Emerging studies show that PKM2 is activated and nuclear translocated in neointimal SMC during neointimal formation. Nuclear PKM2 interacts with STAT3 and promotes STAT3 phosphorylation, thereby regulating MEK5 transcription in SMCs (Jain et al., 2021). At the same time, Liao et al. (Liao et al., 2015) found that STAT3 can regulate SMC phenotypic switching by interacting with myocardin. Furthermore, PKM2 was found to interact with β-catenin and enhance the expression of cyclin D1, GLUT1, and LDHA (Jain et al., 2021). Up-regulation of cyclin D1 and LDHA plays a key role in cell cycle progression and lactate production (Kim et al., 2017). Overexpression of glucose transporter GLUT1 in SMCs mediates their proliferation and phenotypic switching by regulating glucose transport/glucose metabolism (Pyla et al., 2013). It has been found that lactic acid, as a metabolite of glycolysis, can promote the synthetic phenotype of SMCs. Lactate-induced phenotypic modulation of SMCs is mediated by monocarboxylic acid transporters and N-myc downstream regulated gene (Yang et al., 2017). These results indicate that energy metabolism is an important factor influencing SMC functional transformation during tissue ischemia leading to vascular remodeling.

Concerning the molecular mechanisms of SMC phenotype changes, epigenetic inheritance also plays an important role in the process (Alexander and Owens, 2012). Epigenetic inheritance refers to heritable changes in the function of a gene without changes in its DNA sequence, which ultimately lead to changes in the phenotype. Epigenetic regulatory mechanisms include DNA methylation, histone acetylation, and noncoding RNA (Yao et al., 2016; Carter and Zhao, 2021). Increased histone acetyltransferase (HAT) activity stimulated SM22α expression, while increased histone deacetylase (HDAC) inhibited SM22α expression (Gomez et al., 2015). PDGF-BB and oxidized phospholipids induce KLF4 to recruit HDAC in the CArG box region. KLF4, ELK-1 and HDAC form a complex, which reduces the histone H3 acetylation level in the SM22α promoter by binding to G/C repressor elements, resulting in down-regulation of SM22α expression (Salmon et al., 2012). MicroRNA also play a key role in the regulation of SMC phenotype, especially the miR-143/145 cluster, which is a potent promoter for the contraction of SMC phenotype. miR-145 maintained the contractile phenotype of SMCs by inducing the expression of contractile genes, while down-regulating the expression of KLF4 and Elk-1 (Cordes et al., 2009; Lovren et al., 2012). Therefore, the mechanism of SMC phenotype switching involves not only different signal pathways but also epigenetic modification that leads to changes of gene expression profile.

There is an animal model of arterial ligation where artery ischemia occurs. The study has shown that increased expression of CyPA secreted by SMCs leads to intimal hyperplasia by promoting cell proliferation and migration. In ischemic arteries, CyPA can induce the increase of VCAM-1 expression, thereby recruiting inflammatory cells to the vessel wall. Additionally, ROS produced locally by inflammatory cells will further promote the release of CyPA from SMCs, which will form a proinflammatory cycle that promotes vascular remodeling (Satoh et al., 2008). Moreover, activated SMCs synthesize and secrete growth factors, proinflammatory factors and matrix proteins to form an extracellular environment, thereby triggering the infiltration and activation of surrounding proinflammatory cells and progenitor cells and further aggravating intimal hyperplasia (Geary et al., 2002). Concomitantly, it was found that neointimal hyperplasia occurred where there were breaches in the internal elastic lamina, and speculated that elastin disruption would promote neointimal hyperplasia (Lin et al., 2021). Elastin depletion in SMCs promotes their transition from a contractile to a synthetic phenotype (Rebello et al., 2021). What’s more, extracellular matrix glycoprotein biglycan enhances vascular smooth muscle cell proliferation through cdk2- and p27-dependent pathways (Shimizu-Hirota et al., 2004). This may be a reactive response to ischemia, which triggers transcriptional and phenotypic changes in SMCs, causing them to migrate into the intima and proliferate.

**FIGURE 3:**
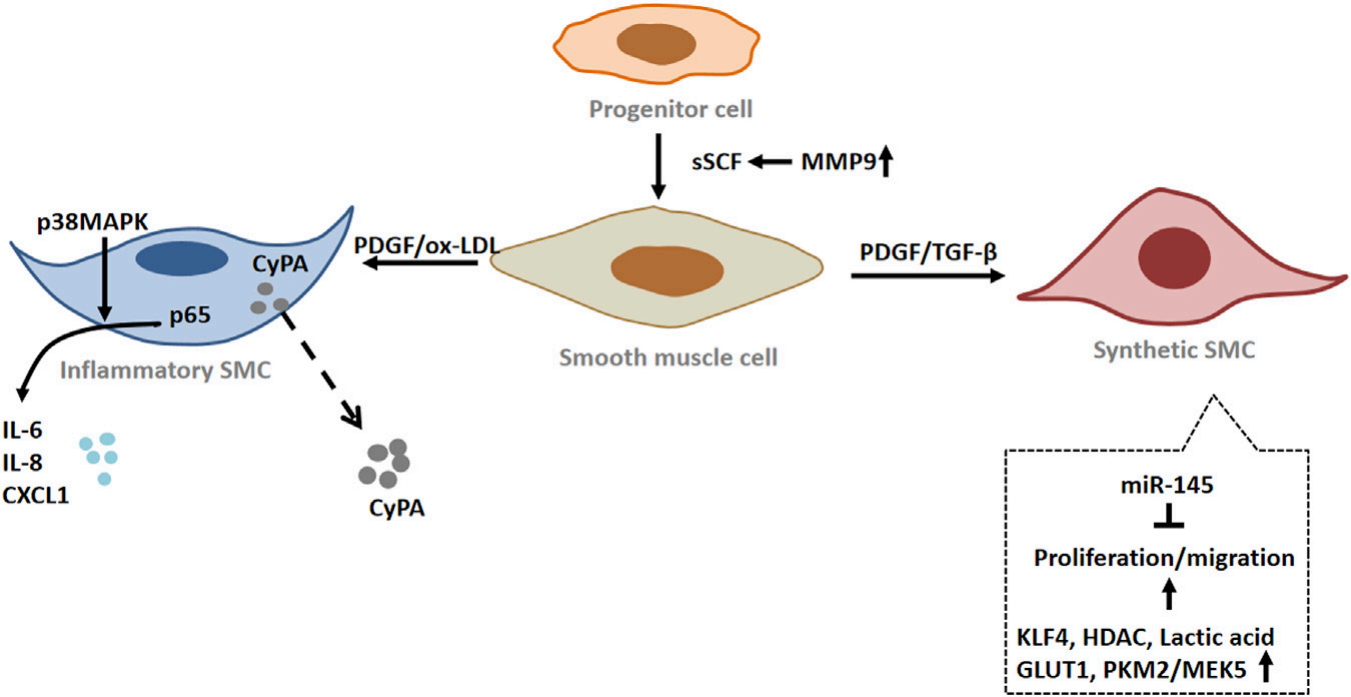
The role of smooth muscle cells (SMCs) in peripheral arterial disease (PAD). Under pathological conditions, smooth muscle cells (SMCs) undergo phenotypic switching. The proliferation and migration of SMC with synthetic phenotype were enhanced. Inflammatory SMCs produce and secrete a variety of inflammatory factors. When blood vessels are injured, vascular progenitor cells become activated and differentiate into SMCs. Abbreviations: SCF, stem cell factor; MMP9, matrix metalloproteinase 9; PDGF, platelet derived growth factor; IL-6, interleukin-6; IL-8, interleukin 8; CXCL1, C-X-C motif chemokine ligand 1; p38 MAPK, p38 mitogen activated protein kinase; HDAC, histone deacetylase; PKM2, pyruvate kinase M2; MEK5, mitogen-activated protein kinase 5; CyPA, cyclophilin A; p65, nuclear factor-kB p65; GLUT1, glucose transporter 1.

As mentioned above, ischemia alters the balance of redox process in the arterial wall. Redox signaling helps maintain the SMC contractile phenotype and proliferative response associated with vascular remodeling (Tong et al., 2010; Tong et al., 2015). As a member of the p38MAPK family, MAPK14 is a redox regulatory element. MAPK14 is considered to be a key regulator of SMC inflammation, proliferation and migration. Previous studies have found that SMC-MAPK14 can regulate MKL1 nuclear translocation and is a negative regulator of SMC contractile phenotype (Long et al., 2013). Some studies showed that in the neointima of mouse carotid arteries, SMCs in the neointima region showed high expression level of MAPK14 protein, accompanied by a decrease in the expression of MYH11. NOX4 is a key mediator of MAPK14 regulatory of SMC contractile gene expression (Wu et al., 2019). At the same time, knockdown of MAPK14 in SMC would lead to down-regulation of PDGFα and PDGFβ expression, thereby inhibiting the proliferation of SMCs. SMC-MAPK14 in neointima activates proinflammatory gene programs through a p65/NF-KB-dependent pathway. MAPK14 phosphorylates p65, which induces the expression of proinflammatory genes such as IL6, IL8, and CXCL1. Interestingly, in the neointimal region, high expression of MAPK14 was also observed in some CD45-positive inflammatory cells in addition to SMCs. The infiltrating inflammatory cells interact with inflammatory SMCs, leaving the vascular in a “hyperinflammatory” state (Wu et al., 2019). These results suggest that high expression of MAPK14 in SMCs in neointimal region regulates the pro-proliferative and pro-inflammatory properties of SMCs through two different pathways. Taken together, these studies suggest that SMCs play a central role in driving lesion development and vascular remodeling via “phenotypic switching” (Figure 3).

Mechanistically, there is a study found that stem cell factor (SCF)/c-Kit tyrosine kinase signaling pathway can regulate neointima formation. The investigators found that vascular injury not only resulted in local upregulation of membrane-bound SCF (mSCF) expression, but also increased circulating SCF (sSCF) levels. SCF not only promotes CD34 ^+^ cell mobilization, but also promotes progenitor adhesion to the exposed matrix on injured vessels, and mediates the differentiation of c-Kit + cells into SMCs (Wang et al., 2006). The researchers found that MMP-9 is activated and cleaves mSCF in response to vascular injury, increasing circulating SCF levels. Some studies have reported that activation of MMP-9 can also promote the release of soluble Kit ligand (sKitL), thus playing a role in progenitor mobilization (Heissig et al., 2002). By using genetic cell lineage tracing techniques, the direct evidence from our group demonstrated that c-Kit + cells can differentiate into SMCs *in vitro* and *in vivo* (Ni et al., 2019). In this aspect, detailed review has been published (Zhang et al., 2018). Thus, progenitor cells may be a source of SMCs during vascular remodelling.

### The role of fibroblasts in peripheral arterial disease

Adventitia is the outermost connective tissue of blood vessels. In recent years, some reports have suggested that the adventitia is a key regulator that directly or indirectly modulates the structure and function of the vascular wall (Patel et al., 2000; Hu and Xu, 2011). Fibroblasts are the most important cell types in the adventitia of blood vessels, which may be the first cells of vascular to be activated in response to ischemia and hypoxia. When being activated, fibroblasts will produce more collagens, secret cytokines and be more actively proliferating (Anwar et al., 2012). Activation of fibroblasts by various stimuli can lead to differentiation of fibroblasts into a myofibroblast phenotype. The expression of α-SMA is an important signal for fibroblasts to differentiate into myofibroblasts. Myofibroblasts are key players in vascular remodeling due to their ability to respond promptly to changes in the local environment *in vivo*, including the production of extracellular matrix proteins, as well as various cytokines and ROS (Gabbiani, 2003). In addition, myofibroblasts can migrate from the adventitia to the media and even to the intima, thereby promoting vascular pathological remodeling (Li et al., 2000).

15-hydroxyeicosaenoic acid (15-HETE) is known as an important factor in vessel remodeling under ischemia/hypoxia, participating in the mediation of cell proliferation, migration and other biological processes (Ma et al., 2011). Zhang et al. (Zhang et al., 2016) found that 15-HETE induced fibroblasts to differentiate into myofibroblasts and promoted FGF-2 expression in adventitial fibroblasts by activating the p38MAPK/EGR-1 pathway. FGF2 not only increased the expression of α-SMA, but also promoted the proliferation of fibroblasts by inhibiting p27kip1 (Zhang et al., 2016). As a CDK inhibitor, p27kip1 negatively regulates the cell cycle by inhibiting cyclin activity and prevents the G1/S phase transition of cells, thereby inhibiting cell proliferation (Goncalves et al., 2015). Other studies have found that FGF-2 mediated cell phenotypic changes and increased nuclear FoxO3a through PI3K-Akt and ERK pathways during hypoxia-induced vascular remodeling (Khalil et al., 2005). At the same time, 15-HETE promotes the migration of adventitial fibroblasts and induces the up-regulation of STAT3 expression in fibroblasts. JNK1-mediated CREB activation was required for 15-HETE-induced STAT3 phosphorylation in VAFs. Phosphorylated STAT3 binds to the MMP2 promoter and induces the expression of MMP-2, thereby promoting the migration and phenotypic changes of fibroblasts (Zhang et al., 2015).

Fibroblast proliferation, migration, differentiation and collagen production are energy-dependent processes. Fatty acid oxidation is a process of energy metabolism that occurs in mitochondria by converting fatty acids into various products [e.g acetyl coenzyme A (acetyl-CoA)] to generate energy (Rodgers et al., 2020). Fatty acid binding protein 3 (FABP3) plays an important role in fatty acid oxidation, controlling fatty acid transport to maintain lipid metabolism and energy homeostasis (Storch and Corsico, 2008). An emerging study showed that FABP3 expression was increased in the adventitia of patients with arteritis and was positively correlated with adventitial fibroblast proliferation and ECM production such as FN1 and collagens (Wu et al., 2022). Elevated serum FABP3 levels have been reported to be associated with increased collagen1 expression and myocardial fibrosis (Maneikyte et al., 2020). Further studies revealed that FABP3 upregulation in adventitial fibroblast promoted the expression of carnitine palmitoyl transferase 1A (CPT1A) and carnitine/acylcarnitine carrier protein (CACT), two key enzymes in FAO, as well as the upregulation of adenosine triphosphate (ATP) (Wu et al., 2022). This suggests that FABP3-mediated progression of vascular fibrosis may be achieved by promoting fatty acid oxidation and ATP production in fibroblasts. Extracellular ATP plays an important role in vascular remodeling. It has been found that hypoxia stimulates the release of exogenous ATP, which can stimulate fibroblast proliferation by autocrine or paracrine pathways (Gerasimovskaya et al., 2002).

**FIGURE 4:**
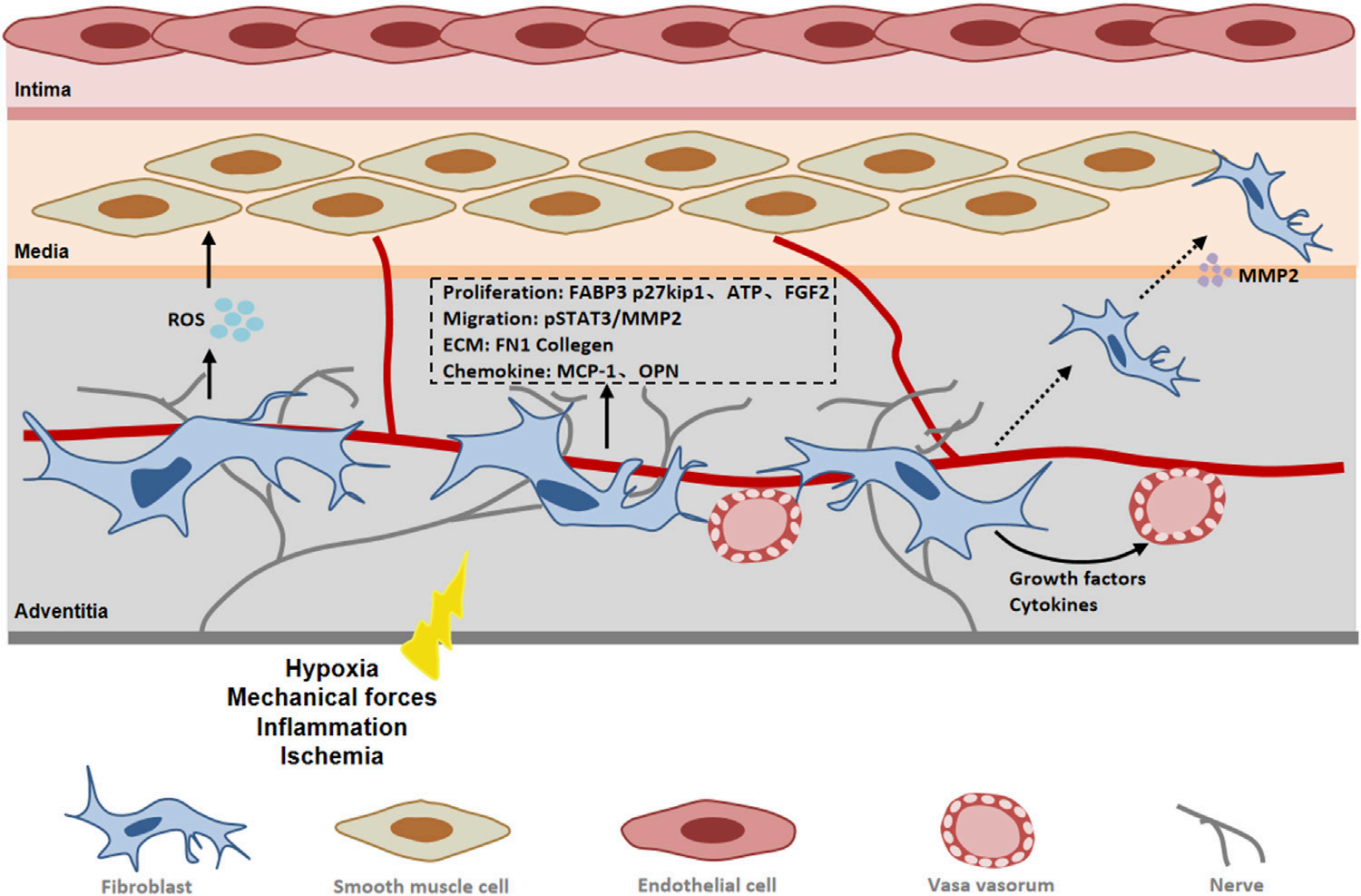
The role of fibroblast in peripheral arterial disease (PAD). In response to vascular injury, adventitial fibroblasts become activated and differentiate into myofibroblasts. Fibroblasts contribute to vascular remodeling through proliferation, migration and extracellular matrix synthesis. Abbreviations: FGF2, fibroblast growth factor 2; FABP3, fatty acid binding protein 3; MMP2, matrix metalloproteinase 2; pSTAT3, phosphorylated signal transducer and activator of transcription 3; ROS, reactive oxygen species; FN 1, fibronectin 1; OPN, osteopontin.

Adventitia plays a crucial role in the initiation and maintenance of vascular inflammation (Moreno et al., 2002). Vascular inflammation has traditionally been considered an inside-out response centered on the recruitment of leukocytes to the intima of vessels. However, a growing body of experimental evidence supports an outside-in hypothesis that vascular inflammation begins and persists in the adventitia and progresses toward the intima (Okamoto et al., 2001). After ischemia/hypoxia, activated adventitial fibroblasts promote the expression of chemokines and adhesion molecules and attract macrophages to the perivascular tissue and adventitia (Hu and Xu, 2011). There is a study revealed that JE/MCP-1 expression was up-regulated in fibroblasts at the early stage of atherosclerosis (Xu et al., 2007). Vascular endothelial growth factor (VEGF) is highly expressed in vascular endothelial cells and exerts its effects mainly through two tyrosine kinase receptors, Flt-1 and Flk-1 (Carmeliet, 2000). It has been found that Flt-1 is increased in adventitial fibroblasts of damaged arteries (Jin et al., 2007). Exogenous VEGF was found to regulate OPN protein expression by promoting ERK1/2 phosphorylation by acting on outer membrane fibroblast Flt-1. AF-derived OPN is a key mediator of VEGF/FLT-1-induced macrophage chemotaxis (Li et al., 2012). OPN can attract inflammatory cells to migrate and adhere to diseased blood vessels, and regulate cytokine production in macrophages, dendritic cells and T cells (Scatena et al., 2007). Further studies demonstrated that antagonistic Flt-1 could inhibit the proliferation of fibroblasts and neointima formation. It has been found that adventitia fibroblasts can migrate through the media to the neointima in damaged blood vessels. This implies that the contribution of Flt-1 to VEGF-mediated neointima formation may be due to inflammatory activation leading to adventitial cell proliferation and migration. In response to inflammatory stimulation, adventitial fibroblasts produce a large amount of NOX-derived ROS. Casino et al. found that adventitia-derived hydrogen peroxide enhanced vasoconstriction by activating p38 MAP kinase in medial SMC as well as inhibiting SHP-2 phosphorylation (Cascino et al., 2011). It was found that fibroblast-NOX2 modulates the growth of SMC in a GDF6-dependent manner. GDF6 is a member of TGF-β superfamily (Harrison et al., 2021) (Figure 4). We hypothesized that in vascular ischemia, leukocytes recruited by adventitial fibroblasts may infiltrate into the media through vasa vasorum, thereby promoting the development of inflammation. At the same time, fibroblasts are associated with peripheral vascular remodeling via secretion of cytokines that influence SMCs and even endothelium.

### General treatment

Improved treatment for PAD must begin with lifestyle interventions, including smoking cessation, diet, and exercise. Smoking is an important risk factor for the development of PAD. It promotes thrombosis through effects on platelets, ECs, and the coagulation system by inducing oxidative stress and lowering nitric oxide (Celermajer et al., 1993; Anand, 2017). Therefore, quitting smoking can reduce the damage to vessels caused by nicotine in cigarettes and slow down the progression of PAD. Epidemiological studies connect healthy dietary patterns with lower incidence of clinical PAD. Improving the quality of diet is important for people with PAD, including fiber and fruit intake (Kulezic et al., 2019). Exercise therapy can maintain and improve the walking ability and quality of life of people with PAD through a series of supervised and systemic exercises (Hamburg and Balady, 2011). Supervised exercise training has been found to improve painless walking distance and maximum walking distance in patients with intermittent claudication (Parmenter et al., 2015). Another trial showed that weekly high-intensity home training, which stimulates moderate-to-severe ischemic leg pain, also significantly improved walking distance (McDermott et al., 2021). This suggests that PAD patients need to make exercise plans according to their own conditions and make timely adjustments to adapt to high-intensity exercise.

### Therapy with drugs

The American College of Cardiology (ACC)/American Heart Association (AHA) recommends high-strength statins for all patients with PAD (Gerhard-Herman et al., 2017). Statin therapy is the most recognized cholesterol-lowering drug in PAD drug therapy. Over the median 5.4-year follow-up period, the incidence of carotid murmurs and claudication was reduced in the simvastatin group (Pedersen et al., 1998). In a retrospective cohort study of diabetic patients with and without PAD, Sohn et al. (2013) compared outcomes in patients taking statins with those taking non-statin lipid-lowering drugs and found that statin therapy reduced amputation rates in patients with PAD. Anticoagulant therapy and antiplatelet therapy are also important to prevent vascular obstruction in patients with PAD. During a median of 23 months of follow-up, the combination of low-dose rivaroxaban and aspirin reduced mortality in patients with PAD as compared with aspirin alone (Eikelboom et al., 2021). In addition, Cilostazol has vasodilatation and antiplatelet function. By inhibiting phosphodiesterase activity in platelets and SMCs, cilostazol can increase the concentration of cAMP in platelets and SMCs and exert antiplatelet and vasodilatation effects (Hashimoto et al., 2006). There is a study showed that cilostazol not only significantly increased the maximum walking distance on the treadmill in PAD patients, but also reduced the risk of amputation (higher risk (HR) 0.42, 95% CI 0.27–0.66) and repeat revascularization (relative risk (RR) 0.44, 95% CI 0.37–0.52) in patients with PAD who underwent revascularization (Desai et al., 2021). Although these drug treatments can improve the symptoms of PAD, it is far from optimal. Understanding the molecular mechanism of the vascular disease development could offer some potential targets for the therapy (Table 1).

**TABLE 1:** Summary of PAD treatment.

Tharepy	Intervention	Effectiveness
General treatment	smoking	Reduced mortality and amputation rates in patients with PAD
	diet	Improve intermittent claudication and severe limb ischemia
	exercise	improve painless walking distance and maximum walking distance
Drugs	statin	Reduce the incidence of intermittent claudication and amputation in patients with PAD
	rivaroxaban/aspirin	Reduce vascular obstruction and the mortality of PAD patients
	cilostazol	Increase the maximum walking distance and reduced the risk of amputation (HR 0.42, 95%CI 0.27–0.66) and repeat revascularization (relative risk (RR) 0.44, 95% CI 0.37–0.52) in patients with PAD

### Summary and perspectives

In summary, the incidence of PAD is increasing year by year, which requires more clinical attention and early intervention. Current studies have shown that multiple mechanisms are involved in peripheral vascular remodeling in PAD, including EndMT, phenotypic transition of SMCs, and activation of fibroblasts. However, the current research is relatively limited, and we need to further explore the underlying mechanism. Firstly, endothelial functional changes in the development of PAD have not been fully understood, although some progress in the field was achieved, including NO/ROS balance, which was used as drugs for treatment of vascular disease. Current techniques of single cell RNA and protein sequencing combining with genetic approaches might be powerful tool for further understanding the mechanisms of cell dysfunctions. Secondly, for SMC biology, most studies focus on phenotype switching from contractile to secretary cells that proliferate and secrete matrix proteins. However, accumulating evidence indicates resident stem/progenitor cells may differentiate into SMCs where they participate in neointima hyperplasia (Wang et al., 2015). Finally, fibroblasts in the vessel wall have been paid attention recently. In this aspect, there is a large amount of work being to do. If researchers in these fields could make studies thoroughly, some major progress for the pathogenesis of PAD may be seen in near future.

At the present, the main treatment methods for PAD are drug therapy and surgery. However, patients with arterial occlusion who cannot undergo revascularization can only undergo amputation. In recent years, great progress has been made in the research of vascular stem cells. Animal studies have demonstrated that vascular stem cells can significantly improve blood supply to ischemic limbs. Novel therapies that promote tissue regeneration and stimulate vasculogenesis and angiogenesis are urgently needed for the treatment of the patients. Cell-based therapies have great potential for the treatment of ischemic diseases (Sprengers et al., 2008; Gupta and Losordo, 2011). Therefore, stem cell therapy could be a potential approach for the treatment of PAD in the future.

## Glossary

PAD: peripheral arterial disease
ASO: atherosclerosis obliterans
SMCs: smooth muscle cells
ECs: endothelial cells
α-SMA: α-smooth muscle actin
HUVECs: human umbilical vein endothelial cells
NO: nitric oxide
EndMT: endothelial-to-mesenchymal transition
AMPK: AMP-activated protein kinase
eNOS: endothelial nitric oxide synthase
ROS: reactive oxygen species
HDAC: histone deacetylase
15-HETE: 15-hydroxyeicosaenoic acid
FABP3: fatty acid binding protein 3
CPT1A: carnitine palmitoyl transferase 1A
CACT: carnitine/acylcarnitine carrier protein
CXCL1: C-X-C motif chemokine ligand 1
PKM2: pyruvate kinase M2
CyPA: cyclophilin A
GLUT1: glucose transporter 1
MMP2: matrix metalloproteinase 2
FN 1: fibronectin 1
ATP: adenosine triphosphate
KLF4: Kruppel like factor 4
TN-X: Tenascin-X
Ox-LDL: oxidized low-density lipoprotein
IL-1β: interleukin 1 β
IL-6: interleukin 6
IL-8: interleukin 8
ICAM-1: intercellular cell adhesionmolecule-1
VCAM-1: vascular cell adhesion molecule-1
MCP-1: monocyte chemotactic protein-1
FoxM1: Forkhead Box M1
ET-1: endothelin-1
SCF: stem cell factor
MMP9: matrix metalloproteinase 9
PDGF: platelet derived growth factor
TGF-β: transforming growth factor-β
p38 MAPK: p38 mitogen activated protein kinase
MEK5: mitogen-activated protein kinase 5
p65: nuclear factor-kB p65
FGF2: fibroblast growth factor 2
pSTAT3: phosphorylated signal transducer and activator of transcription 3
OPN: osteopontin
